# Supplementary material for: Approximate Bayesian computation supports a high incidence of chromosomal mosaicism in blastocyst-stage human embryos
Source: Genetics. 2025 Aug 1;231(2):iyaf149. doi: 10.1093/genetics/iyaf149 (PMC12505293; doi:10.1093/genetics/iyaf149)
Supplement: iyaf149_Supplementary_Data [file iyaf149_supplementary_data.zip › Supplementary_Figure_1_GENETICS-2025-308243.pdf]

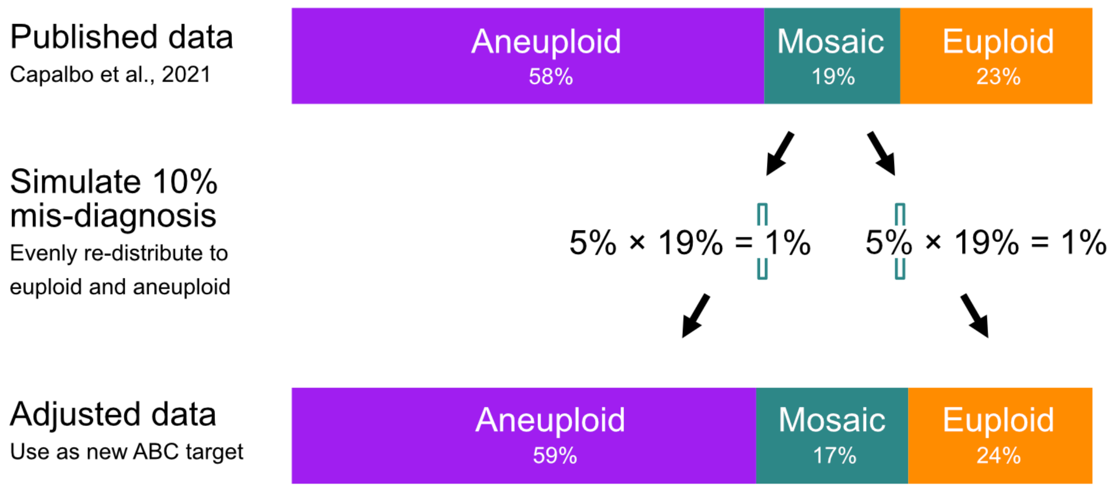

Supplementary Figure 1: Schematic of approach for simulating misclassification of mosaic embryos. We simulated varying levels of misclassification spanning from 0% to 100% in 10% increments. At each misclassification level, half of the mis-classified mosaic biopsies were assumed to be euploid and the other half aneuploid. These adjusted values were used as the new targets for ABC. The figure depicts an example of simulating a misclassification rate of 10% (Capalbo et al. 2021).
