# Supplementary material for: Approximate Bayesian computation supports a high incidence of chromosomal mosaicism in blastocyst-stage human embryos
Source: Genetics. 2025 Aug 1;231(2):iyaf149. doi: 10.1093/genetics/iyaf149 (PMC12505293; doi:10.1093/genetics/iyaf149)
Supplement: iyaf149_Supplementary_Data [file iyaf149_supplementary_data.zip › Supplementary_Table_1_GENETICS-2025-308243.pdf]

	Dispersal = 0		Dispersal = 0.5		Dispersal = 1	
	**Meiotic**	**Mitotic**	**Meiotic**	**Mitotic**	**Meiotic**	**Mitotic**
**Mean**	0.40	0.063	0.57	0.021	0.58	0.015
**Pctl. 2.5**	0.34	0.054	0.54	0.018	0.55	0.014
**Pctl. 25**	0.38	0.059	0.56	0.020	0.57	0.015
**Pctl. 50**	0.40	0.062	0.57	0.020	0.58	0.015
**Pctl. 75**	0.42	0.065	0.58	0.021	0.59	0.016
**Pctl. 97.5**	0.45	0.071	0.60	0.023	0.60	0.017
**MAP**	0.40	0.063	0.57	0.020	0.58	0.015

Supplementary Table 1: Inferred rates of meiotic and mitotic error at varying levels of dispersal. The reported statistics are summaries of the corresponding posterior distributions. The 2.5 and 97.5 percentiles of the posterior distributions form the boundaries of the 95% credible intervals. The last row reports the maximum *a posteriori* (MAP) estimate (Capalbo et al. 2021).
