# Supplementary material for: Approximate Bayesian computation supports a high incidence of chromosomal mosaicism in blastocyst-stage human embryos
Source: Genetics. 2025 Aug 1;231(2):iyaf149. doi: 10.1093/genetics/iyaf149 (PMC12505293; doi:10.1093/genetics/iyaf149)
Supplement: iyaf149_Supplementary_Data [file iyaf149_supplementary_data.zip › Supplementary_Table_2_GENETICS-2025-308243.pdf]

	Dispersal = 0			Dispersal = 0.5			Dispersal = 1		
	**Euploid**	**Mosaic**	**Aneuploid**	**Euploid**	**Mosaic**	**Aneuploid**	**Euploid**	**Mosaic**	**Aneuploid**
**Mean**	0.23	0.19	0.58	0.23	0.19	0.58	0.23	0.19	0.58
**Std. Dev.**	0.0043	0.0031	0.0047	0.0032	0.0054	0.0056	0.0027	0.0048	0.0049
**Min.**	0.22	0.18	0.57	0.23	0.18	0.57	0.23	0.18	0.57
**Pctl. 25**	0.23	0.18	0.58	0.23	0.18	0.58	0.23	0.18	0.58
**Pctl. 50**	0.23	0.19	0.58	0.23	0.19	0.58	0.23	0.19	0.58
**Pctl. 75**	0.23	0.19	0.58	0.23	0.19	0.58	0.23	0.19	0.58
**Max.**	0.24	0.19	0.59	0.24	0.20	0.59	0.24	0.20	0.59

Supplementary Table 2: Biopsy results for the simulations selected by ABC. For all simulated levels of dispersal, the biopsy results closely matched the targets from the published data, supporting the model fit (Capalbo et al. 2021).
