# Supplementary material for: Approximate Bayesian computation supports a high incidence of chromosomal mosaicism in blastocyst-stage human embryos
Source: Genetics. 2025 Aug 1;231(2):iyaf149. doi: 10.1093/genetics/iyaf149 (PMC12505293; doi:10.1093/genetics/iyaf149)
Supplement: iyaf149_Supplementary_Data [file iyaf149_supplementary_data.zip › Supplementary_Table_3_GENETICS-2025-308243.pdf]

Dispersal	Total aneuploid biopsies	Number of aneuploid biopsies originating from mosaic embryos
0	581518	183313 (31.5%)
0.5	581303	7891 (1.4%)
1	583075	3975 (0.7%)

Supplementary Table 3: Proportion of aneuploid biopsies (i.e., biopsies where all 5 sampled cells were aneuploid) obtained from embryos that were actually mosaic. All embryos used for biopsy were from the posterior predictive samples generated from the distribution of meiotic and mitotic error rates selected at each dispersal level.
