# Supplementary material for: Approximate Bayesian computation supports a high incidence of chromosomal mosaicism in blastocyst-stage human embryos
Source: Genetics. 2025 Aug 1;231(2):iyaf149. doi: 10.1093/genetics/iyaf149 (PMC12505293; doi:10.1093/genetics/iyaf149)
Supplement: iyaf149_Supplementary_Data [file iyaf149_supplementary_data.zip › Supplementary_Table_4_GENETICS-2025-308243.pdf]

| Embryo type | First biopsy | Second biopsy | Dispersal = 0 | Dispersal = 0.5 | Dispersal = 1 |
|---|---|---|---|---|---|
| Mosaic aneuploid | Euploid | Euploid | 108408 (47%) | 150107 (64.9%) | 156474 (68%) |
| Mosaic aneuploid | Euploid | Mosaic | 67793 (29.4%) | 80452 (34.8%) | 73288 (31.9%) |
| Mosaic aneuploid | Euploid | Aneuploid | 54450 (23.6%) | 702 (0.3%) | 49 (0%) |
| Mosaic aneuploid | Mosaic | Euploid | 70813 (37.7%) | 79995 (42.7%) | 77195 (41.3%) |
| Mosaic aneuploid | Mosaic | Mosaic | 59526 (31.7%) | 102154 (54.5%) | 106954 (57.2%) |
| Mosaic aneuploid | Mosaic | Aneuploid | 57492 (30.6%) | 5272 (2.8%) | 2804 (1.5%) |
| Mosaic aneuploid | Aneuploid | Euploid | 55148 (9.5%) | 759 (0.1%) | 72 (0%) |
| Mosaic aneuploid | Aneuploid | Mosaic | 57261 (9.8%) | 5410 (0.9%) | 2891 (0.5%) |
| Mosaic aneuploid | Aneuploid | Aneuploid | 70904 (12.2%) | 1722 (0.3%) | 1012 (0.2%) |
| Fully aneuploid | Aneuploid | Aneuploid | 398205 (68.5%) | 573412 (98.6%) | 579100 (99.3%) |
| Fully euploid | Euploid | Euploid | NA | 15 (0%) | 161 (0.1%) |

**Supplementary Table 4: First and second biopsy summaries of embryos constructed from the posterior distributions of meiotic and mitotic error rates inferred with ABC.** At each dispersal level, we also report the percentages of each combination of embryo type, first biopsy type, and second biopsy type out of all embryos of a given first biopsy type
