# Supplementary material for: Approximate Bayesian computation supports a high incidence of chromosomal mosaicism in blastocyst-stage human embryos
Source: Genetics. 2025 Aug 1;231(2):iyaf149. doi: 10.1093/genetics/iyaf149 (PMC12505293; doi:10.1093/genetics/iyaf149)
Supplement: iyaf149_Supplementary_Data [file iyaf149_supplementary_data.zip › Supplementary_Table_5_GENETICS-2025-308243.pdf]

Data set	Maternal Age
Capalbo et al. (2021)	Mean: 38.06<br>Standard deviation: 3.65
Clarke et al. (2023)	Median: 34.0<br>670 patients < 35<br>462 patients > 35
Munné et al. (2017)	Mean: 35.8
Rodrigo et al. (2020)	Mean: 38.6<br>Standard deviation: 6.2

Supplementary Table 5: Maternal ages of PGT-A patients across input datasets. Studies differed in reported measures of central tendency and variability.
